# Lifetime stress accelerates epigenetic aging in an urban, African American cohort: relevance of glucocorticoid signaling

**DOI:** 10.1186/s13059-015-0828-5

**Published:** 2015-12-17

**Authors:** Anthony S. Zannas, Janine Arloth, Tania Carrillo-Roa, Stella Iurato, Simone Röh, Kerry J. Ressler, Charles B. Nemeroff, Alicia K. Smith, Bekh Bradley, Christine Heim, Andreas Menke, Jennifer F. Lange, Tanja Brückl, Marcus Ising, Naomi R. Wray, Angelika Erhardt, Elisabeth B. Binder, Divya Mehta

**Affiliations:** Department of Translational Research in Psychiatry, Max Planck Institute of Psychiatry, Munich, Germany; Department of Psychiatry and Behavioral Sciences, Duke University Medical Center, Durham, NC USA; Institute of Computational Biology, Helmholtz Zentrum München, Neuherberg, Germany; Department of Psychiatry and Behavioral Sciences, Emory University Medical School, Atlanta, GA USA; Howard Hughes Medical Institute, Chevy Chase, MD USA; Yerkes National Primate Research Center, Emory University, Atlanta, GA USA; Department of Psychiatry and Behavioral Sciences and the Center on Aging, University of Miami Miller School of Medicine, Miami, FL USA; Atlanta Veterans Affairs Medical Center, Decatur, GA USA; Institute of Medical Psychology, Charité Universitätsmedizin Berlin, Berlin, Germany; Current Address: Department of Psychiatry, Psychosomatics, and Psychotherapy, University of Wuerzburg, Wuerzburg, Germany; Max Planck Institute of Psychiatry, Munich, Germany; The University of Queensland, Queensland Brain Institute, St Lucia, Qld 4072 Australia; Department of Biobehavioral Health, Pennsylvania State University, University Park, PA USA

**Keywords:** Aging, Aging-related disease, DNA methylation, Epigenetics, Gene expression, Glucocorticoids, Psychological stress

## Abstract

**Background:**

Chronic psychological stress is associated with accelerated aging and increased risk for aging-related diseases, but the underlying molecular mechanisms are unclear.

**Results:**

We examined the effect of lifetime stressors on a DNA methylation-based age predictor, epigenetic clock. After controlling for blood cell-type composition and lifestyle parameters, cumulative lifetime stress, but not childhood maltreatment or current stress alone, predicted accelerated epigenetic aging in an urban, African American cohort (n = 392). This effect was primarily driven by personal life stressors, was more pronounced with advancing age, and was blunted in individuals with higher childhood abuse exposure. Hypothesizing that these epigenetic effects could be mediated by glucocorticoid signaling, we found that a high number (n = 85) of epigenetic clock CpG sites were located within glucocorticoid response elements. We further examined the functional effects of glucocorticoids on epigenetic clock CpGs in an independent sample with genome-wide DNA methylation (n = 124) and gene expression data (n = 297) before and after exposure to the glucocorticoid receptor agonist dexamethasone. Dexamethasone induced dynamic changes in methylation in 31.2 % (110/353) of these CpGs and transcription in 81.7 % (139/170) of genes neighboring epigenetic clock CpGs. Disease enrichment analysis of these dexamethasone-regulated genes showed enriched association for aging-related diseases, including coronary artery disease, arteriosclerosis, and leukemias.

**Conclusions:**

Cumulative lifetime stress may accelerate epigenetic aging, an effect that could be driven by glucocorticoid-induced epigenetic changes. These findings contribute to our understanding of mechanisms linking chronic stress with accelerated aging and heightened disease risk.

**Electronic supplementary material:**

The online version of this article (doi:10.1186/s13059-015-0828-5) contains supplementary material, which is available to authorized users.

## Background

The last decades have witnessed a dramatic increase in life expectancy. As a result, the number of older adults is predicted to more than double over the next two decades [1, 2]. While this increase in life expectancy is undoubtedly one of the biggest achievements of modern medicine, population aging also brings forth an unprecedented increase in aging-related diseases, including cardiovascular disease, cancer, and dementia [3]. Given that these conditions are currently the leading causes of morbidity and mortality, it is imperative to gain insights into factors that impact healthy aging and contribute to aging-related diseases.

An important risk factor for accelerated aging and aging-related diseases is psychological stress. Although stressors are ubiquitous in nature and necessary for survival [4], excessive and chronic stress has been associated with accelerated cellular aging [5, 6] and increased risk for aging-related disease phenotypes, including cardiovascular disease, immune dysregulation, and late-life neuropsychiatric disorders [7–12]. Furthermore, stressors occurring during sensitive developmental periods, such as childhood maltreatment, have been linked with later development of aging-related diseases [13–15]. Lastly, stress-related psychiatric disorders, including major depression and post-traumatic stress disorder (PTSD), are themselves risk factors for such diseases [15, 16]. Despite these observations, the molecular mechanisms linking psychological stress with accelerated aging and aging-related diseases remain largely unknown.

One plausible mechanism that may mediate the adverse effects of stress on the aging process is epigenetic regulation. Long-term epigenetic changes can be induced by environmental stimuli, including psychological stressors, and can shape complex phenotypes [17]. The most studied epigenetic modification in this context is DNA methylation. Stressors can induce lasting changes in DNA methylation [18, 19], an effect that is in part mediated by the genomic effects of glucocorticoids, a primary molecular effector of the stress response [20]. Glucocorticoids exert actions in essentially every body organ via activation of the glucocorticoid receptor (GR), a transcription factor that regulates gene expression by the binding of its homodimer to glucocorticoid response elements (GREs) in regulatory regions of target genes [21]. Beyond regulating gene transcription, GRE binding can locally induce lasting changes in DNA methylation, a form of molecular memory that shapes subsequent responses to glucocorticoids and stressors [17, 18, 22–24]. Therefore, it is plausible that stress and glucocorticoid exposure throughout the lifetime could impact cellular aging via cumulative effects on aging-related DNA methylation sites.

Aging and aging-related diseases are associated with profound changes in DNA methylation [25–31]. Recognizing the importance of DNA methylation in the aging process has led to recent development of several DNA methylation-based predictors of aging [27, 32–34]. Among these, a composite predictor comprised of 353 Cytosine-phosphate-Guanosine sites (CpGs) across the genome (‘epigenetic clock’) was shown to strongly correlate with chronological age across multiple tissues in humans [27], suggesting its usefulness as a biomarker in aging-related research. Using this predictor, accelerated epigenetic aging (Δ-age), defined as the difference between DNA methylation-predicted age (DNAM-age) and chronological age, has been associated with aging-related and other phenotypes, including cancer, obesity, cytomegalovirus infection, Down’s syndrome, PTSD, physical and cognitive decline, all-cause mortality, and the presence of higher self-control and lower socioeconomic status [27, 35–41]. However, no studies have examined the relationship between this predictor and cumulative lifetime stress nor the potential molecular mechanisms underlying this relationship.

In the present study, we first show that cumulative lifetime stress, but not childhood or current stress alone, is associated with accelerated epigenetic aging in a cohort of highly traumatized African American individuals. Examining GR signaling as a potential mechanism underlying this effect, we identify that a high number of epigenetic clock CpGs are located within functional GREs and show dynamic methylation changes following GR activation by exposure to the GR agonist dexamethasone (DEX). Lastly, we show that genes neighboring these CpGs are dynamically regulated by DEX and that these DEX-regulated genes show enriched association for aging-related diseases. Taken together, our findings support a model of stress-induced acceleration of epigenetic aging, overall contributing to our understanding of mechanisms linking chronic stress with accelerated aging and heightened disease risk.

## Results

### Prediction of chronological age using the epigenetic clock

**Fig. 1:**
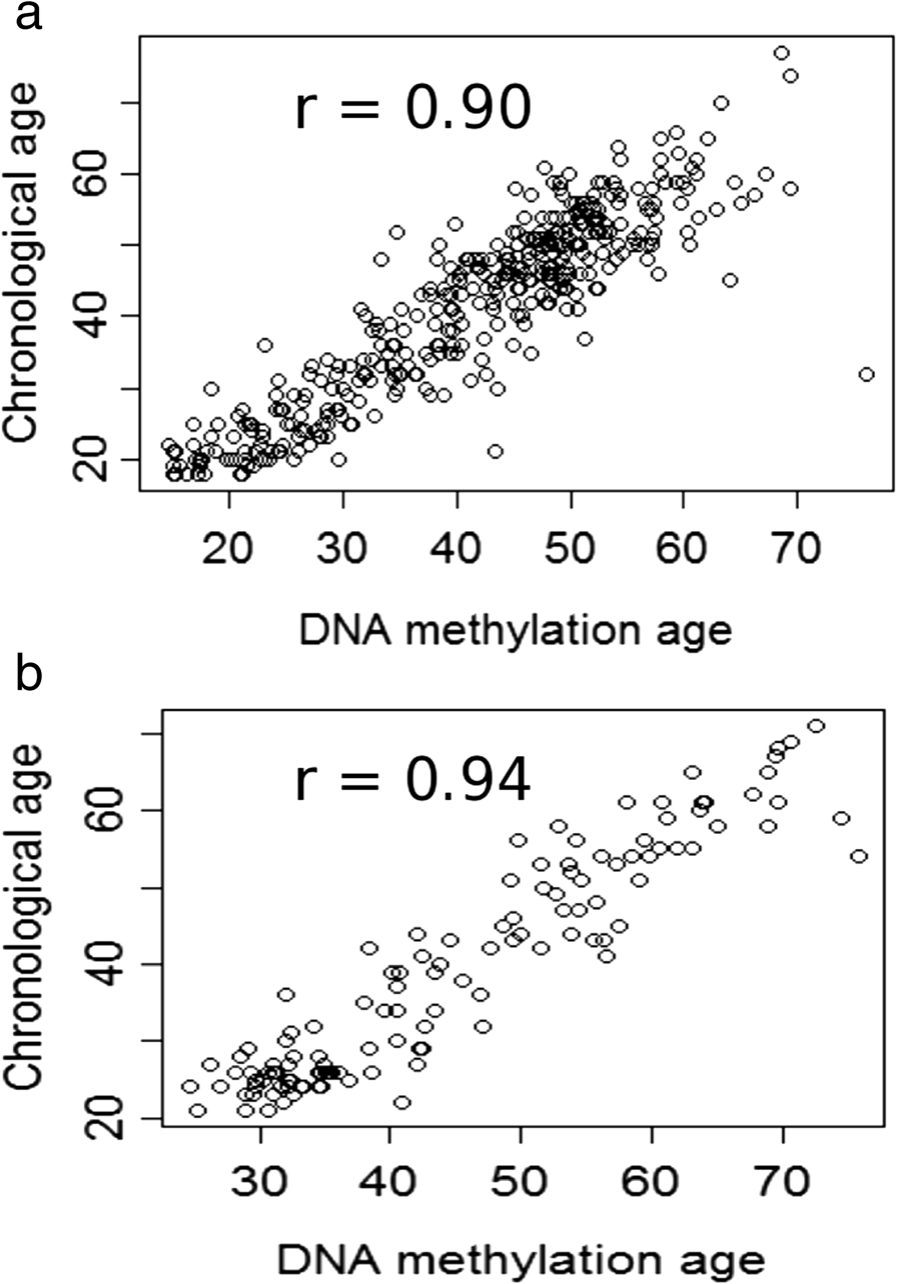
Correlation between chronological age and age predicted by DNA methylation-based predicted age in two independent cohorts. **a** GTP cohort (n = 393). **b** MPIP cohort (n = 124)

DNAM-age was calculated from peripheral blood from two independent samples, derived from the Grady Trauma Project (GTP) and the Max Planck Institute of Psychiatry (MPIP) cohorts using genome-wide Illumina HumanMethylation450 BeadChips (450 K), as previously described [27]. Given that the GTP primarily comprises (>90 %) African American participants, we excluded other ethnicities to minimize confounders. This resulted in a total of 393 participants with DNAM-age data. In contrast, the MPIP cohort consists only of Caucasian participants with a total of 124 participants with baseline DNAM-age data. The mean (SD, range) age was 41.33 years (12.85, range 18 to 77 years) for the GTP and 39.5 years (14.14, range 21 to 71 years) for the MPIP. The n (%) of female participants was 278 (70.7 %) for the GTP and 44 (35.5 %) for the MPIP. To validate the epigenetic clock predictor in our cohorts, we correlated DNAM-age with chronological age as previously described [27]. This correlation was strong for both the GTP (*r* = 0.90, *P* <2.2 × 10^−16^) (Fig. 1a) and MPIP (*r* = 0.94, *P* <2.2 × 10^−16^) cohorts (Fig. 1b) and proved robust and similar for both genders (*r* = 0.89 for male vs. *r* = 0.90 for female in the GTP; *r* = 0.95 for male vs. *r* = 0.94 for female in the MPIP).

### Epigenetic age acceleration is associated with cumulative lifetime stress, but not childhood or current stress alone, in an urban, African American cohort

**Fig. 2:**
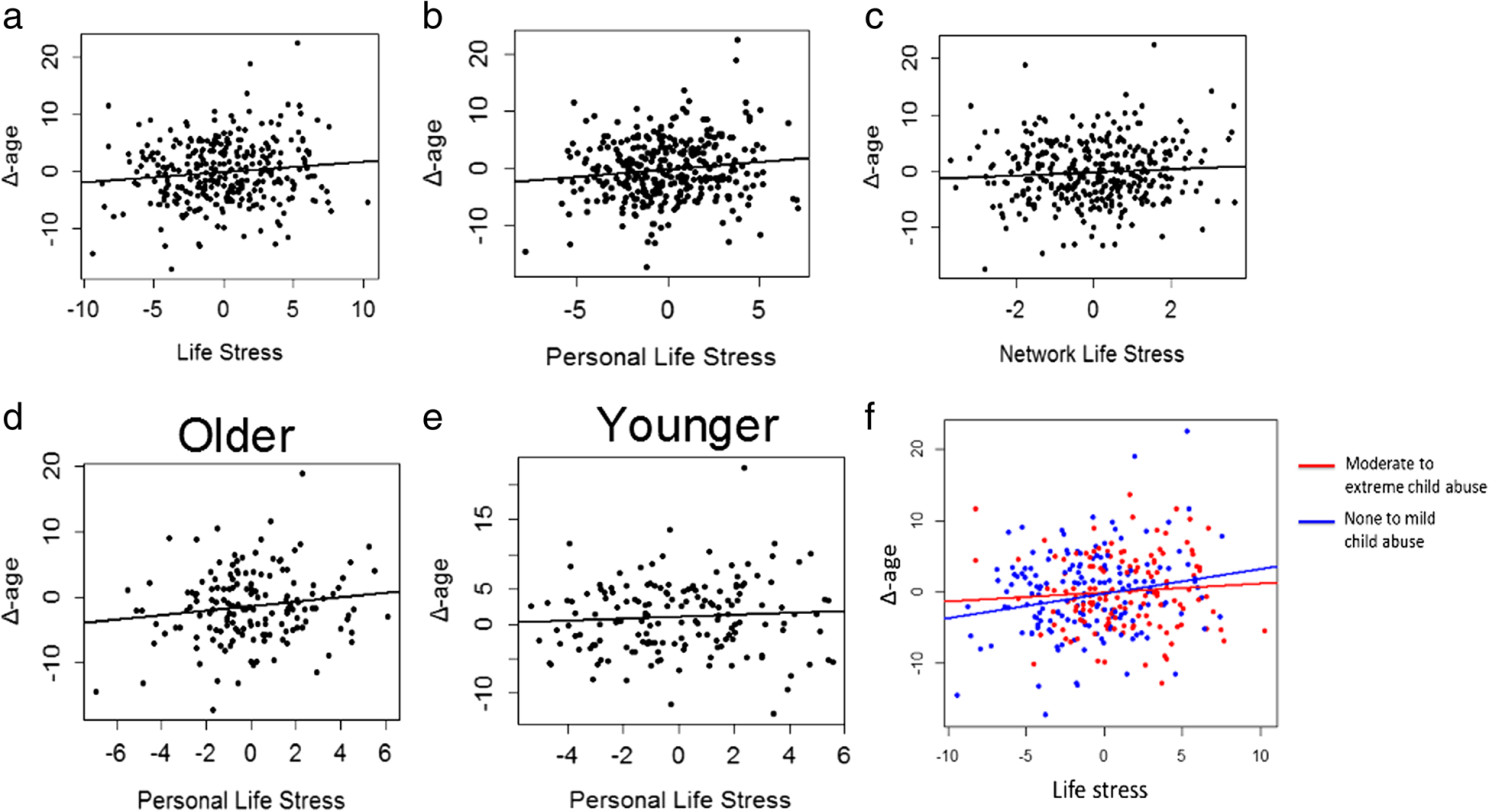
Cumulative lifetime stress is associated with epigenetic age acceleration in a highly traumatized human cohort derived from the Grady Trauma Project. Epigenetic age acceleration (Δ-age) was calculated by subtracting chronological age from DNA methylation predicted age. Δ-age was regressed on cumulative lifetime stress (Life Stress) after adjusting for covariates (fitted stress measures are shown). **a** Life Stress was positively associated with epigenetic age acceleration (β = 0.18, SE = 0.08, *P* = 1.8 × 10^−2^), and this association remained significant after further controlling for lifestyle parameters, including body mass index, smoking, alcohol, cocaine, marijuana, and heroin use (β = 0.31, SE = 0.11, *P* = 7.4 × 10-3), as well depressive symptomatology, psychiatric treatments, and genome-wide SNP-based principal components (β = 0.28, SE = 0.13, *P* = 2.7 × 10^−2^). Statistically significant association was found for Personal Life Stress (β = 0. 26, SE = 0.10, *P* = 8.7 × 10^−3^) (**b**), whereas the effect of Network Life Stress was not significant (*P* = 1.1 × 10^−1^) (**c**). Age stratification by a median split showed that the effect of Personal Life Stress on Δ-age was stronger in older (β = 0.33, SE = 0.17, *P* = 5.3 × 10^−2^) (**d**), as compared to younger participants (β = 0.15, SE = 0.14, *P* = 2.8 × 10^−1^) (**e**). Stratification of the effect of cumulative life stress on epigenetic age acceleration based on the presence or not of moderate to severe physical or sexual child abuse showed that Life Stress was positively associated with Δ-age in participants with no or mild physical and sexual child abuse (β = 0.34, SE = 0.11, *P* = 2.5 × 10^−3^, n = 212) but not in those with moderate to extreme child abuse (*P* = 3.9 × 10^−1^, n = 174) (**f**)

We then hypothesized that epigenetic age acceleration (Δ-age), calculated by subtracting the actual chronological age from DNAM-age [27], would be positively associated with exposure to life stress. This hypothesis was tested in the highly traumatized GTP cohort. The mean (SD, range) Δ-age in the GTP was –0.13 years (5.69, range –17.31 to 43.98 years). A total of 304 GTP participants had data on lifetime stressors assessed by the Stressful Events Questionnaire (SEQ) and 386 participants had data on childhood maltreatment assessed by the Childhood Trauma Questionnaire (CTQ). The individual items from the SEQ were summed to yield a total score of lifetime stress exposure (Life Stress), and a similar total score was generated for the CTQ (Child Stress). The SEQ additionally assesses stressor exposure over the last year, and these items were summed to yield a score of more recent stress exposure (Current Stress). Linear regression models controlling for sex and age showed that Life Stress was positively associated with Δ-age (β = 0.24, SE = 0.08, *P* = 2.8 × 10^−3^), and this effect remained significant after further controlling for Houseman blood cell counts and technical batch effects (β = 0.18, SE = 0.08, *P* = 1.8 × 10^−2^) (Fig. 2a), lifestyle parameters, including body mass index, smoking, alcohol, cocaine, marijuana, and heroin use (β = 0.31, SE = 0.11, *P* = 7.4 × 10^−3^), as well depressive symptomatology, psychiatric treatments, and genome-wide SNP-based principal components (β = 0.28, SE = 0.13, *P* = 2.7 × 10^−2^).

In secondary analyses, we examined whether the effect of lifetime stress on age acceleration depends on the type of stressor and other moderating variables. Based on previous work distinguishing between life events that affect the individual directly vs. life events that affect one’s social network [42], we separately summed SEQ items assessing personal life events (Personal Life Stress) and items assessing network events (Network Life Stress). Δ-age showed a positive and significant association with Personal Life Stress (β = 0.26, SE = 0.10, *P* = 8.7 × 10^−3^) (Fig. 2b) and a positive but not significant association with Network Life Stress (*P* = 1.1 × 10^−1^) (Fig. 2c). No significant interactions were noted between Life Stress or Personal Life Stress and either sex or age. However, stratification of the GTP by a median split of age showed that the effect of Personal Life Stress on Δ-age was marginally stronger in older (β = 0.33, SE = 0.17, *P* = 5.3 × 10^−2^) (Fig. 2d) as compared to younger participants (β = 0.15, SE = 0.14, *P* = 2.8 × 10^−1^) (Fig. 2e). On the other hand, Δ-age was not associated with either CTQ score (*P* = 4 × 10^−1^) or Current Stress alone (*P* = 1.3 × 10^−1^). However, when participants were stratified based on the severity of childhood maltreatment, only individuals exposed to lower levels (none or mild) of sexual and physical childhood abuse (based on respective CTQ subscale scores) showed significant effects of Life Stress on Δ-age (Fig. 2f). This was not a consequence of differential stress exposure burden between the two groups, since, as expected, individuals exposed to higher levels of childhood abuse also had higher levels of Life Stress with a mean (SD) Life Stress of 12.32 (3.64) as compared to 10.01 (3.76) in individuals with lower levels of childhood abuse (t_299_ = 5.38, *P* = 1.5 × 10^−7^). Furthermore, the two strata showed similar correlations between DNAM-age and chronological age (*r* = 0.91 for higher vs. 0.92 for lower abuse, Fisher z score = 0.6, *P* = 5.5 × 10^−1^). Lastly, we found no association between Δ-age and current stress-related psychiatric phenotypes, including depressive (*P* = 3.4 × 10^−1^) and PTSD symptomatology (*P* = 7.9 × 10^−1^) in the GTP. In line with this finding, depression diagnosis was not associated with Δ-age in the MPIP cohort (*P* = 2.3 × 10^−1^, n = 72 controls vs. 52 depressed). Taken together, these findings show that cumulative lifetime stress, but not childhood trauma or current stress alone, is associated with accelerated epigenetic aging, an effect that is primarily driven by personal life events, may be more evident in advancing ages, and is blunted in participants exposed to high levels of childhood abuse.

### Epigenetic clock CpGs and neighboring genes are regulated by GR activation and show enriched association with aging-related diseases

The effect of lifetime stress on epigenetic aging prompted us to examine susceptibility of individual epigenetic clock CpGs to glucocorticoids, a primary molecular effector of stress responses, as a potential mechanism underlying this association. To address this hypothesis, we first examined whether epigenetic clock CpGs show DNA methylation changes 3 h after oral exposure to a GR agonist (1.5 mg of DEX) in the independent MPIP cohort (n = 124). After correcting for multiple testing, 110 of the 353 CpGs showed statistically significant methylation changes (false discovery rate (FDR)-adjusted *P* <5 × 10^−2^). Among the DEX-regulated CpGs, 98 (89 %) showed decrease in methylation, whereas 12 (11 %) showed increase in methylation (Additional file 1: Table S1). We next examined the effect of acute DEX exposure on the epigenetic clock by comparing DNAM-age at baseline vs. 3 h after DEX exposure (n = 124). There was no effect of DEX on DNA methylation-predicted age (baseline mean DNAM-age = 45.24 vs. post-DEX mean DNAM-age = 45.15, paired t_123_ = 0.31, *P* = 7.6 × 10^−1^).

**Fig. 3:**
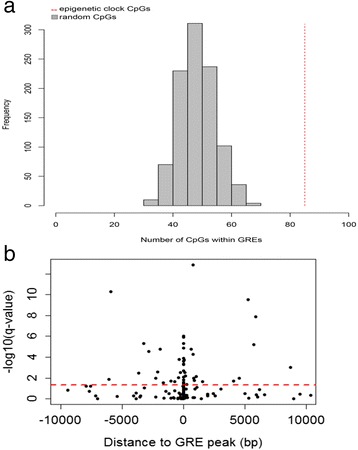
Epigenetic clock CpGs co-localize with functional glucocorticoid response elements (GREs) and show methylation changes following GR activation. **a** Epigenetic clock CpGs co-localize with functional GREs. GRE peaks were derived from ENCODE *NR3C1* ChIP-seq data from lymphoblastoid cell lines. Among the 353 epigenetic clock CpGs, 85 CpG sites were noted to be located within GR ChIP-Seq peaks in a lymphoblastoid cell line (shown with the red dotted line) (Additional file 1: Table S1). This number significantly differed (p_perm_ <0.001) from the CpG-GRE overlap predicted by 1,000 randomly selected sets of CpGs covered by the 450 K array (mean 48.8, SD 6.14, range 31 to 68). **b** Epigenetic clock CpGs that are significantly regulated by DEX exposure are in proximity to GREs. GRE peaks were derived from ENCODE *NR3C1* ChIP-seq data from lymphoblastoid cell lines. Volcano plot was zoomed for +/− 10 kb distance around the GRE peaks. The dotted red line in the volcano plot represents the level of statistical significance (*P* = 5 × 10^−2^) after FDR correction for multiple comparisons. Further details on DEX-regulated CpGs are given in Additional file 1: Table S1

Given that GR binding to GREs can exert changes in DNA methylation, we then examined whether epigenetic clock CpGs co-localize with GREs. Among the 353 epigenetic clock CpGs, 85 CpGs were located within GREs as defined by CHIP-Seq peaks in a lymphoblastoid cell line (LCL) (Additional file 1: Table S1). This CpG-GRE co-localization significantly differed from the one expected by chance as determined by randomly drawing 1,000 sets (n = 353 CpGs) of CpG sites from all CpGs present on the 450 K array (expected mean 48.8, SD 6.1, range 31 to 68, p_perm_ <1 × 10^−3^) (Fig. 3a). Proximity to GREs was particularly observed for DEX-regulated CpGs (Fig. 3b), with 17 of these sites located right within GREs and 35 within 1 kb distance from GREs. Because the 353 CpGs were originally derived from the 21,369 (21 K) CpGs that overlap the 27 K and 450 K Illumina arrays [27], we next examined whether the epigenetic clock CpG-GRE co-localization differs from the one present in the 21 K background. Epigenetic clock CpG-GRE co-localization did not differ from the one expected by chance when randomly drawing 1,000 CpG sets (n = 353 CpGs) from the 21 K CpG sites (expected mean 3,094, SD 50.7, range 2,927–3,270, p_perm_ = 9.7 × 10^−1^). Given that this lack of enrichment could be the result of high CpG-GRE co-localization already present in the 21 K, as a last step we compared the co-localization present in the 21 K with the 450 K background and we noted significantly higher CpG-GRE co-localization in the 21 K as compared to the 450 K background (p_perm_ <1 × 10^−3^). These findings suggest that the increased epigenetic clock CpG-GRE co-localization is a more general property of the 21 K CpGs used to develop the epigenetic clock. Yet the presence of a high number of epigenetic clock CpGs within functional GREs is in line with our hypothesis that these sites may be highly susceptible to GR activation.

**Fig. 4:**
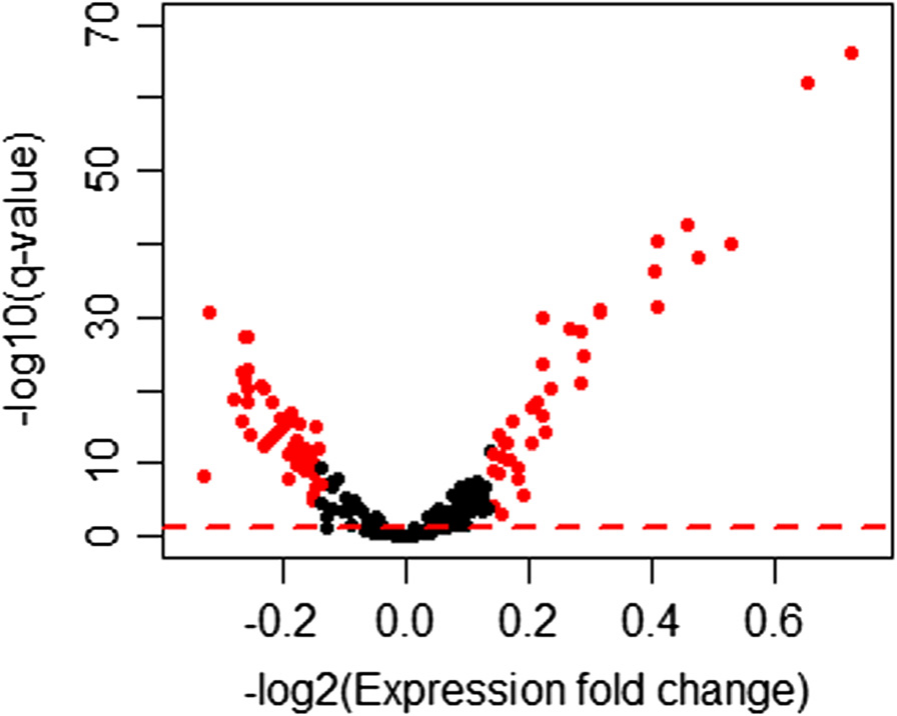
Glucocorticoid receptor activation regulates the expression of genes with transcription start sites (TSS) near epigenetic clock CpGs. Gene TSS near epigenetic clock CpGs were identified based on the annotation from [43]. The volcano plot shows DEX-induced fold change in gene expression plotted against their corrected *P* values (q values). The dotted red line represents the corrected level of statistical significance (q = 5 × 10^−2^) after FDR correction for multiple comparisons. Among the 216 unique array probes, 167 probes, corresponding to 139 unique genes, showed significant changes in gene expression following DEX. Fifty-eight per cent of these probes (n = 97) showed upregulation and 42 % (n = 70) showed downregulation. The mean (SD, range) distance of each regulated gene TSS to the corresponding epigenetic clock CpGs was ±419.3 bp (336.65 bp, range 1 to 1,423 bp). Marked in red are the probes showing fold changes in gene expression >1.1. Further details are provided in Additional file 2: Table S2

We then assessed whether genes that have transcription start sites (TSS) in the proximity of epigenetic clock CpGs are also dynamically regulated by GR activation. For this purpose, we used peripheral blood genome-wide gene expression array data in the MPIP cohort to examine the DEX-induced changes in the expression of genes with transcription start sites (TSS) close to epigenetic clock CpGs based on the 450 K annotation from [43]. Using these criteria, we annotated 344 unique genes. Of these, 333 genes were present on the gene expression microarray and a total of 170 genes, corresponding to 220 epigenetic clock CpGs, were expressed above background in the MPIP cohort (Additional file 2: Table S2). Transcription of these genes was detected by 216 unique gene expression array probes. After FDR-based correction for multiple testing, 167 out of the 216 detected probes, corresponding to 139 unique genes (81.7 %), showed significant changes in gene expression following DEX exposure (FDR-adjusted *P* values <0.05) (Fig. 4). Fifty-eight per cent of these probes (n = 97) showed upregulation and 42 % (n = 70) showed downregulation. The mean (SD, range) distance of each regulated gene TSS to the corresponding epigenetic clock CpGs was ±419.3 bp (336.65 bp, range 1 to 1,423 bp). To rule out potential bias derived from the 21 K background, we then asked whether genes neighboring epigenetic clock CpGs are more responsive to GR activation compared to genes neighboring the 21 K CpGs. A total of 5,443 unique genes, corresponding to 21,015 21 K CpGs, showed significant DEX-induced mRNA expression changes (FDR-adjusted *P* values <5 × 10^−2^). The number of DEX-regulated genes was significantly higher for the genes with TSS close to epigenetic clock CpGs as compared to 21 K CpGs (Fisher’s exact test *P* = 6.3 × 10^−5^). Taken together, these data demonstrate enhanced responsivity of genes neighboring epigenetic clock CpGs to GR activation.

Lastly, we performed disease enrichment analysis in WebGestalt using the set of unique DEX-regulated genes (n = 139) as the input for the analysis and the genes expressed above background in our peripheral blood gene expression arrays as the reference set of genes. After FDR correction for multiple testing, this resulted in enriched association for aging-related diseases, including coronary artery disease, arteriosclerosis, and leukemias (FDR-adjusted *P* <5 × 10^−2^ each) (Additional file 3: Table S3).

## Discussion

The present study sought to determine the effect of life stressors on epigenetic aging, as measured with the epigenetic clock [27] in peripheral blood samples. While previous studies found associations of the epigenetic clock with several phenotypes [27, 35–41], this is the first study to use this predictor in a highly traumatized cohort. As hypothesized, accelerated epigenetic aging was associated with cumulative lifetime stress burden. Given that epigenetic effects of the stress response can be mediated by GR signaling, we further examined the molecular basis of this association by annotating epigenetic clock CpG sites in relation to GREs and examining the impact of GR activation on these sites. We found that GREs co-localize with epigenetic clock CpGs and that glucocorticoid activation can induce dynamic methylation changes of these sites as well as changes in the expression of genes neighboring epigenetic clock CpGs. Taken together, these converging findings support a model of stress-induced accelerated epigenetic aging, plausibly mediated by the lasting effects of cumulative stressor exposure and aberrant glucocorticoid signaling on the epigenome.

Further examination of the relationship between life stress and epigenetic aging led us to several interesting observations. First, this relationship was apparent for cumulative stress exposure throughout the lifetime, whereas no significant association was found with childhood maltreatment or current stress alone. This finding is in accordance with a recent study observing no effect of childhood trauma on epigenetic aging in combat veterans [35] and suggests that cumulative stressors over the lifetime, rather than time-limited stressors either during childhood or adulthood, have a stronger or more lasting effect on epigenetic aging. Nonetheless, it is also possible that these null findings may be due to lack of power, the timing of DNA methylation assessments, or reversibility of epigenetic aging, possibilities that could be addressed by future longitudinal studies. Second, the effect of lifetime stress was driven by personal stressors – affecting the participant directly – rather than network stressors that occur to someone within the participant’s network. This is congruent with previous studies showing that personal life events are more strongly correlated with genetic factors as compared to network events [44]. In line with the effects of lifetime vs. current stress, these effects were more pronounced in older individuals, suggesting cumulative epigenetic vulnerability in older individuals. Lastly, the epigenetic effects of lifetime stress were blunted in individuals with higher levels of childhood abuse. This finding could not be attributed to differences in the levels of lifetime stress, since individuals exposed to higher levels of childhood abuse also had higher levels of cumulative lifetime stress burden. Thus, it is possible that early trauma exposure triggers additional mechanisms of risk and resilience that may interfere with subsequent effects of stressors on epigenetic aging, a hypothesis that remains to be tested by future studies.

The effects of lifetime stress on epigenetic aging in peripheral blood are likely mediated by persistent neuroendocrine alterations induced by cumulative stress exposure. Stressors and glucocorticoids can drive persistent changes in the expression of glucocorticoid-responsive genes and concomitant changes in DNA methylation at CpGs located at or near GREs [17, 18, 22, 45]. Supporting this hypothesis, we noted that a high number of epigenetic clock CpG sites are located within functional GREs and show dynamic methylation changes following DEX exposure. Notably, most of these CpGs show DEX-induced decrease in methylation, whereas far fewer sites show increased methylation (98 vs. 12). This is in accordance with previous studies showing that activation of the GR results in local demethylation of CpGs in the proximity of a GRE [18, 22, 23] and that site-specific decreases in methylation have been implicated in aging-related phenotypes [46]. CpG demethylation has been proposed to be potentially mediated by at least two enzymatic processes, base excision repair and oxidation [47, 48]. Examining the role of these processes may provide further insights into mechanisms of stress-induced epigenetic aging. Furthermore, an open question concerns the sequence of molecular events that determine whether some stress-induced DNA methylation changes become embedded and longlasting, while other changes are dynamic and reversible. Given the low dose and acute exposure to glucocorticoids in our study, additional experiments with different doses and more chronic *in vitro* or *in vivo* GR activation will be necessary to better elucidate this mechanism.

An important implication of our findings is the potential role of stress-induced epigenetic aging in health and disease. Increasing age and aging-related diseases have been associated with global and site-specific changes in DNA methylation [25–30, 39]. The age-related epigenetic clock CpGs co-localize with genes that show enrichment for cell growth and survival, organismal development, and cancer [27]. Furthermore, we show that DEX-regulated genes neighboring epigenetic clock CpGs show enriched association for aging-related diseases, including coronary artery disease, arteriosclerosis, and leukemias. These findings raise the possibility that lifetime stress may contribute to these diseases via its cumulative impact on epigenetic regulation of genes implicated in aging-related diseases.

The findings of the present study should be viewed in the context of its limitations. Although we observe an association between epigenetic age acceleration and lifetime stressors in the GTP cohort, the cross-sectional design of the study limits conclusions regarding the direction of causality. As discussed above, it is plausible that epigenetic aging of peripheral blood cells results from persistent alterations of the neuroendocrine but also immune milieu induced by repetitive stressor exposure. However, accelerated epigenetic aging might alternatively represent a vulnerability marker that predisposes individuals to expose themselves to stressful environments. It is also important to acknowledge that, while the high levels of traumatic events in the GTP make this cohort highly suitable for examining the effects of lifetime stress on the epigenome, they may also limit generalizability of these findings to other less traumatized cohorts. Moreover, the present study examined epigenetic aging in peripheral blood only. While this tissue is easily accessible and relevant for biomarker research, other tissues may be more susceptible to psychological stress and should be examined in the context of specific diseases. For example, disease-specific effects on the epigenetic clock have been demonstrated for liver tissue in the context of obesity [39]. Another limitation is the use of Chip-Seq data from lymphoblastoid cell lines to examine epigenetic clock CpG-GRE co-localization. This cell line represents the best available proxy for peripheral blood, the source tissue for our methylation data, but this approach may also be limited by the tissue specificity of functional GREs and the altered epigenetic landscapes of immortalized cell lines. Lastly, although we corrected for several confounders that might influence DNA methylation, such as sex, age, smoking, body mass index, substance abuse, current psychiatric symptoms and treatments, other factors not captured by our methods may have confounded the observed relationships. These limitations may be overcome in future studies by employing detailed prospective measurements of lifestyle factors, stressor exposure, DNA methylation, and incidence of stress-related phenotypes at different time points throughout the lifetime.

## Conclusions

The present study provides evidence that cumulative life stress exposure is associated with accelerated epigenetic aging and that these effects may be mediated by glucocorticoid signaling. Our findings further suggest that DNA methylation-based age prediction in peripheral blood may be a useful molecular marker to incorporate in future studies examining the effects of life stress exposure. These findings offer novel insights into the molecular mechanisms linking psychological stress with diseases of the aging.

## Methods

### Clinical samples

The effect of lifetime stress on epigenetic aging was examined in the Grady Trauma Project (GTP), a large study conducted in Atlanta, Georgia, that investigates the role of genetic and environmental factors in shaping responses to stressful life events. The GTP includes more than 7,000 participants from a predominantly African American, urban population of low socioeconomic status [49, 50]. This population is characterized by high prevalence and severity of trauma over the lifetime and is thus particularly relevant for examining the effects of stressors on epigenetic markers. For this purpose, we used a subsample of GTP participants with genome-wide DNA methylation data. All participants provided written informed consent and all procedures were approved by the Institutional Review Boards of the Emory University School of Medicine and Grady Memorial Hospital (IRB00002114).

We examined glucocorticoid-induced methylation changes of epigenetic clock CpGs and responsivity of genes closest to these CpGs in 297 Caucasian participants recruited at the Max Planck Institute of Psychiatry (MPIP). Recruitment strategies and characterization of participants have been previously described [51, 52]. These consisted of 200 male (83 healthy probands and 117 inpatients with depressive disorders) and 97 female (48 healthy probands and 49 depressed) individuals. Baseline whole blood samples were obtained at 18:00 after 2 h of fasting and abstention from coffee and physical activity (baseline). Participants then received 1.5 mg oral dexamethasone (DEX) and a second blood draw was performed at 21:00, 3 h after DEX ingestion (post-DEX). The study was approved by the local ethics committee (approval number: 318/00) and all individuals gave written informed consent. All experimental methods comply with the Helsinki Declaration.

### Psychometric instruments

Childhood trauma was measured in the GTP with the Childhood Trauma Questionnaire (CTQ), a validated self-report questionnaire that assesses five types of maltreatment during childhood: sexual, physical, and emotional abuse, as well as emotional and physical neglect [53]. Scores for each type of maltreatment were derived from participant responses to questionnaire items and scores from all types were summed to yield a total CTQ score reflecting overall burden of childhood maltreatment. Moderate to extreme sexual abuse was defined by a cutoff score of 8 or above in the CTQ sexual abuse subscale, and moderate to extreme physical abuse was defined by a cutoff score of 10 or above in the physical abuse subscale as previously described [54].

Stressful lifetime events in the GTP were assessed with the Stressful Events Questionnaire (SEQ), a 39-item self-report instrument that has been described in detail [55]. The SEQ covers a wide range of stressor exposure, ranging from personal life events, such as divorce, unemployment, crime, and financial stressors, to network life events, such as knowing someone who was murdered. Participants report whether they have experienced these events either in the past year or at any time in their life. Although the SEQ assesses life event exposure throughout the lifetime, it does not include questions specific for childhood maltreatment. Life events are summed to yield a total score that reflects the number of stressors experienced over the last year (Current Stress) or cumulative number of stressors experienced throughout one’s lifetime (Life Stress).

Participants underwent the Structured Clinical Interviews for DSM-IV defined psychiatric diagnoses. Given the observed relation between stress-related psychiatric disorders and accelerated cellular aging, we also examined major depression and PTSD as variables of interest. In the GTP, current depressive symptomatology was assessed with the 21-item validated Beck Depression Inventory (BDI) [56, 57] and current PTSD symptomatology was assessed with the validated 17-item PTSD Symptom Scale (PSS) [49, 58].

### DNA methylation

Genomic DNA from the GTP cohort (n = 393) and the MPIP (n = 124) was extracted from whole blood using the Gentra Puregene Blood Kit (QIAGEN). DNA quality and quantity was assessed by NanoDrop 2000 Spectrophotometer (Thermo Scientific) and Quant-iT Picogreen (Invitrogen). Genomic DNA was bisulfite converted using the Zymo EZ-96 DNA Methylation Kit (Zymo Research) and DNA methylation levels were assessed for >480,000 CpG sites using the Illumina HumanMethylation450 BeadChip array. Hybridization and processing was performed according to manufacturer’s instructions as previously described [59]. Quality control of methylation data, including intensity read outs, filtering (detection *P* value >0.01 in at least 75 % of the samples), cellular composition estimation, as well as beta and M-value calculation was done using the minfi Bioconductor R package version 1.10.2 [60].

For the GTP cohort, X chromosome, Y chromosome, and non-specific binding probes were removed [61]. We also excluded probes if single nucleotide polymorphisms (SNPs) were documented in the interval for which the Illumina probe is designed to hybridize. Given that the GTP cohort includes individuals from different ethnicities, we also removed probes if they were located close (10 bp from query site) to a SNP which had Minor Allele Frequency of ≥0.05, as reported in the 1,000 Genomes Project, for any of the populations represented in the samples. Technical batch effects were identified by inspecting the association of the first principal components of the methylation levels with plate, sentrix array, and position (row) and by further visual inspection of principal component plots using the shinyMethyl Bioconductor R package version 0.99.3 [62]. This procedure identified row and slide as technical batches. The raw methylation data and all related phenotypes for the GTP cohort have been deposited into NCBI GEO (GSE72680).

For the MPIP cohort, filtered beta values were reduced by eliminating any CpG sites/probes on sex chromosomes, as well as probes found to have SNPs at the CpG site itself or in the single-base extension site with a MAF ≥1 % in the 1,000 Genomes Project European population and/or non-specific binding probes according to [61]. Additionally, we performed a re-alignment of the array probe sequences using Bismark (doi: 10.1093/bioinformatics/btr167). This yielded a total of 425,883 CpG sites for further analysis. Using the same procedure for batch identification as above, we identified processing (experiment) date as technical batch in the MPIP. The data were then normalized with functional normalization [63], an extension of quantile normalization included in the minfi R package and batch-corrected using ComBat. The raw methylation data and all related phenotypes for the MPIP cohort have been deposited into NCBI GEO (GSE74414).

### Gene expression

In the DEX-treated (MPIP) cohort (n = 297, including the 124 individuals used for the MPIP methylation analysis), both baseline and post-DEX whole blood RNA was collected using PAXgene Blood RNA Tubes (PreAnalytiX), processed as described previously [51, 52]. Samples had a mean RNA integrity number (RIN) of 8 ± 0.51 SD. Blood RNA was hybridized to Illumina HumanHT-12 v3 and v4 Expression BeadChips (Illumina, San Diego, CA, USA). Raw probe intensities were exported using Illumina’s GenomeStudio and further statistical processing was carried out using R. All 29,075 probes present on both microarrays, excluding X and Y chromosomes as well as cross-hybridizing probes identified by using the Re-Annotator pipeline (http://dx.doi.org/10.1101/019596) were first filtered with an Illumina detection *P* value of 0.05 in at least 50 % of the samples, leaving 11,994 expressed probes for further analysis. Subsequently, each transcript was transformed and normalized through variance stabilization and normalization (VSN) [64]. Using the same procedure for batch identification as for the methylation data, we identified slide, amplification round, array version, and amplification plate column as technical batches. The data were then adjusted using ComBat [65] and have been deposited into NCBI GEO (GSE64930).

### Statistical analyses

All statistical analyses were conducted in R version 3.1.0 (http://www.r-project.org/) [66]. Unless indicated otherwise, *P* values are nominal and two-tailed. All corrections for multiple testing were performed using the FDR method of Benjamini and Hochberg. The level of statistical significance was set a priori at 0.05 (5 × 10^−2^).

DNA methylation-based age prediction was performed using the R code and statistical pipeline developed by Horvath [27]. This predictor was developed using 82 Illumina DNA methylation array datasets (n = 7,844) involving 51 healthy tissues and cell types [27]. The raw data were normalized using BMIQ normalization method [67] implemented in the Horvath DNA methylation-based age predictor R script [27]. Robustness and reproducibility of the epigenetic age predictor was tested using 40× technical replicates of an individual control sample, randomized across microarray chips and batches used to measure DNA methylation in the GTP cohort. The average epigenetic age (DNAM-age) of the control sample (true age = 32 years) was 32.64 (SD: 0.23) years with an average correlation *r* = 0.97 (0.001). Age acceleration (Δ-age) was defined (as previously) as the average difference between DNAM-age and chronological age. One GTP participant had extreme Δ-age (43.98 years), and using the Grubbs’ test (http://graphpad.com/quickcalcs/grubbs2/) was noted to be the only outlier (Z = 3.80, *P* <5 × 10^−2^). Although primary analyses were conducted without this outlier, inclusion of this individual did not substantially alter the reported results. Generalized linear regression models tested the relationship of Δ-age with stressors and stress-related phenotypes (GTP cohort). Because DNAM-age is calculated from raw beta values (before Combat correction for batches), technical batches identified for the GTP (row and slide) and the MPIP cohort (processing date) were tested as potential confounders in the respective regression models. In the GTP, models were further adjusted for age, sex, Houseman cell counts, body mass index, smoking, alcohol, current substance abuse, and the principal components from population stratification checks. In the MPIP, models were adjusted for gender, age, body mass index, and Houseman cell counts.

To determine if methylation signals or gene expression levels are significantly different before and after DEX stimulation in the MPIP cohort, likelihood ratio tests accounting for gender, age, body mass index, disease status, and estimated cell-type counts were applied to each CpG site (n = 353) and expression array probe (n = 11,994), respectively. DNA methylation and gene expression changes were corrected for multiple comparisons using FDR. The 353 epigenetic clock CpGs were annotated to a total of 344 genes. Among these, 170 genes were detected in peripheral blood by 216 gene expression array probes (163 genes were expressed below background and 11 genes were not covered by the gene expression arrays).

To account for population stratification due to discrepancies between self-reported and actual race in the GTP, we used genome-wide SNP data that were available for 382 participants. Of the 700 k SNPs present on the Omni Quad and Omni express arrays, 645,8315 autosomal SNPs were left after filtering with the following criteria: minor allele frequency of >1 %; Hardy-Weinberg equilibrium of 0.000001; and genotyping rate of >98 %. The samples were clustered to calculate rates of identity by descent (IBD). We then ran multidimensional scaling analysis on the IBD matrix using PLINK2 (https://www.cog-genomics.org/plink2) and plotted the first ten axes of variation against each other. No outliers were detected. The first two principal components were used as covariates in regression models to adjust for population stratification.

To identify whether epigenetic clock CpG sites are co-localized with GREs, we used ENCODE *NR3C1* ChIP-Seq data from lymphoblastoid cell lines (*accession: ENCSR904YPP*) for which no aligned tracks are currently available. Initial filtering was performed using FASTX Toolkit (v. 0.0.14, http://hannonlab.cshl.edu/fastx_toolkit/index.html) and Prinseq (v. 0.20.3) [68] to eliminate artefacts and low quality reads. Alignment on hg19 was performed using BWA (v. 0.7.10) [69] allowing only uniquely mappable alignments with an alignment quality of above 20. Reads from both ChIP-Seq and both control libraries were pooled leading to 46,453,650 and 68,227,580 used reads, respectively. Peak-calling was carried out by MACS14 (v. 1.4.2) [70] using default settings, resulting in approximately 23,000 annotated signals. The average length of ChIP-Seq signal as defined by the peak calling was 746.3 bps (SD: 370.6). We generated 1,000 sets (n = 353 CpGs) of randomly drawn CpG sites (without replacement) from the set of all CpGs present on the 450 K BeadChip array (excluding X and Y chromosomes). For every set we counted the percentage of CpG sites within a GRE ChIP-Seq signal (+/− 0 bp). On this basis we constructed the null distribution and compared it to the observed percentage of clock CpG sites within a GRE ChIP-Seq signal to measure the enrichment statistics.

Disease enrichment analysis was performed using the WEB-based GEne SeT AnaLysis Toolkit (WebGestalt; http://bioinfo.vanderbilt.edu/webgestalt/) [71, 72]. This was performed by using as input the set of unique DEX-regulated genes neighboring epigenetic clock CpGs (n = 139) and as reference the set of genes expressed above background in our peripheral blood gene expression arrays. The minimum number of genes for the enrichment analysis was set at 5, the statistic performed was hypergeometric test, and results were corrected for multiple testing using FDR.

## Additional files

Additional file 1: Table S1. Location of epigenetic clock CpGs in relation to the nearest glucocorticoid response element (as shown by within GR ChIP-Seq peaks in a lymphoblastoid cell line) and their methylation changes in response to the glucocorticoid receptor agonist dexamethasone. (DEX). (XLSX 68 kb) Additional file 2: Table S2. Annotation of genes with transcription start sites (TSS) near epigenetic clock CpGs and their expression changes in response to DEX. Gene annotation was based on [43]. (XLSX 26 kb) Additional file 3: Table S3. WebGestalt Disease enrichment analysis of the set of unique DEX-regulated genes (n = 139) with TSS near epigenetic clock CpGs. For the primary analysis, we used as reference the set of genes expressed above background in our peripheral blood gene expression arrays. This analysis was repeated using a more condensed background comprised only of the genes neighboring 21 K CpGs that showed DEX-induced mRNA expression changes (n = 5,443). While this post-hoc analysis yielded no statistically significant results after correction for multiple testing (*P* values presented in the last column), the top 10 diseases were very similar (with higher but nominally significant *P* values for the top three hits) with the analysis using the broader reference set of genes. (XLSX 10 kb)
