# Correction to: Lifetime stress accelerates epigenetic aging in an urban, African American cohort: relevance of glucocorticoid signaling

**DOI:** 10.1186/s13059-018-1441-1

**Published:** 2018-05-23

**Authors:** Anthony S. Zannas, Janine Arloth, Tania Carrillo-Roa, Stella Iurato, Simone Röh, Kerry J. Ressler, Charles B. Nemeroff, Alicia K. Smith, Bekh Bradley, Christine Heim, Andreas Menke, Jennifer F. Lange, Tanja Brückl, Marcus Ising, Naomi R. Wray, Angelika Erhardt, Elisabeth B. Binder, Divya Mehta

**Affiliations:** 1 Department of Translational Research in Psychiatry, Max Planck Institute of Psychiatry, Munich, Germany; 2 Department of Psychiatry and Behavioral Sciences, Duke University Medical Center, Durham, USA; 3 Institute of Computational Biology, Helmholtz Zentrum München, Neuherberg, Germany; 4 Department of Psychiatry and Behavioral Sciences, Emory University Medical School, Atlanta, USA; 5 Howard Hughes Medical Institute, Chevy Chase, USA; 6 Yerkes National Primate Research Center, Emory University, Atlanta, USA; 7 Department of Psychiatry and Behavioral Sciences and the Center on Aging, University of Miami Miller School of Medicine, Miami, USA; 8 Atlanta Veterans Affairs Medical Center, Decatur, USA; 9 Institute of Medical Psychology, Charité Universitätsmedizin Berlin, Berlin, Germany; 10 Present Address: Department of Psychiatry, Psychosomatics, and Psychotherapy, University of Wuerzburg, Wuerzburg, Germany; 11 Max Planck Institute of Psychiatry, Munich, Germany; 12 The University of Queensland, Queensland Brain Institute, St Lucia, Australia; 13 Department of Biobehavioral Health, Pennsylvania State University, University Park, USA

## Erratum

Upon publication of the original article [1] it was highlighted by the authors that a transposition error affected Additional file 1, causing the misplacement of several columns and rendering the table difficult to read. This transposition does not influence any of the results nor analyses presented in the paper and has since been formally noted in this correction article; the corrected file is available here as an Additional File. The publisher apologizes for this error.

## Additional file

Additional file 1: Location of epigenetic clock CpGs in relation to the nearest glucocorticoid response element (as shown by within GR ChIP-Seq peaks in a lymphoblastoid cell line) and their methylation changes in response to the glucocorticoid receptor agonist dexamethasone. (XLSX 69 kb)
